# Modeling Soil Water Dynamics and Hydrogel Doses Optimization Using a Machine Learning Approach: A Case Study on Sandy Clay Loam Soil under Varying Bulk Densities

**DOI:** 10.1021/acsomega.5c05318

**Published:** 2025-11-24

**Authors:** José Wilson de Oliveira Magalhães, Ednaldo José Ferreira, Líllian Alexia Lameira da Rocha, José Manoel Marconcini, Carlos Manoel Pedro Vaz, Luís Henrique Bassoi

**Affiliations:** † College of Agricultural Science, Department of Agricultural Engineering, São Paulo State University, Av. Universitária 3780, 18610-307 Botucatu, SP, Brazil; ‡ Embrapa Instrumentation, Rua XV de Novembro 1452, 13561-206 São Carlos, SP, Brazil

## Abstract

Climate change has intensified droughts in Brazil, threatening agriculture through altered rainfall and temperature patterns. A promising approach to mitigating the soil water deficit is the addition of biodegradable hydrophilic polymers (hydrogels). However, water dynamics in soil hydrogel systems remain complex and depend on soil type, bulk density, and hydrogel dosage. The hydrophilic properties of the matrix may persist over time, highlighting the importance of hydrogel residual effects. The influence of bulk density on polymer dosage dynamics remains underexplored, and no rapid analytical method currently exists to estimate the dose equivalence of active hydrogels for agricultural practices with accuracy and without excessive time consumption. This study addresses two main goals: (1) to evaluate the effectiveness of hydrogel dosages in sandy clay loam soil at varying densities for enhanced water retention and (2) to develop a machine learning-based analytical method for rapid estimation of active hydrogel doses from short soil water content time series. Results showed a significant increase in soil water retention at the 3 g L^–1^ dosage. The proposed method, using a locally weighted regression model, achieved a high correlation (0.875) and low error (0.749 g L^–1^) in cross-validation without requiring density information, offering a practical tool for agricultural applications. These findings support the efficient and sustainable use of hydrogels, providing a practical framework that facilitates their management and enables rapid field-scale interventions to improve water use in agriculture.

## Graphical abstract

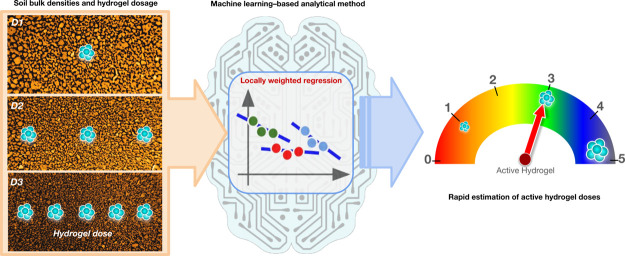

## Introduction

In recent years, climate change has impacted several regions of Brazil, with a particular emphasis on semiarid areas. Changes in rainfall patterns and air temperature have led to drought periods that significantly affect agricultural production. These irregularities in weather events have negatively impacted the socioeconomic conditions of many Brazilian regions. Rural communities are increasingly challenged to build resilience in their agricultural systems in the face of water scarcity and irregular rainfall distribution and volume. Agriculture in these areas raises concerns regarding the use of water (irrigation) in comparison to other sectors, such as energy generation (hydroelectric plants) and services, or even for residential and industrial purposes.^1^

Strategies and actions at local and regional scales for agricultural adaptation to climate change have been based on crop varieties and management, land use changes, and innovative breeding techniques; water and soil management practices; and farmer training and knowledge transfer.^2^ In terms of soil and water management, hydrogels, also known as hydrophilic (superabsorbent) polymers, have gained traction as a promising solution for improving water availability for crops due to their properties of water absorption, biodegradability, water retention, and slow-release capacity.^3,4^

Hydrogels consist of a three-dimensional (3D) network of polymer chains with some portions solvated by water molecules and others chemically or physically linked. This structure gives them the property of swelling without dissolving in an aqueous environment.^5^ These cross-linked polymers can absorb hundreds to thousands of times their dry weight and retain large amounts of water without dissolving due to their 3D structured network and hydrophilic functional groups, such as amine (−NH2), carboxylic acid (−COOH), sulfate (−SO3H), and hydroxyl (−OH) groups. For agricultural purposes, their slow-release characteristics can reduce the need for irrigation water, improve nutrient utilization efficiency, and reduce water pollution.^6–9,4^ The addition of the hydrogel to the soil matrix modifies and extends its original hydraulic and structural properties.

The soil texture influences changes promoted by incorporating hydrogels into this porous medium, deriving a new soil hydrogel matrix.^10–12^ High soil temperatures can affect the efficacy of some types of hydrogels, reducing their absorption capacities.^13–15^ Hydrogels alter the basic physical properties of soil as a result of their strong hydrophilicity and the changes in volume that occur during wetting and drying cycles.^16^ They can also reduce the soil bulk density, infiltration rate, water diffusivity, saturated hydraulic conductivity, and penetration resistance. In contrast, hydrogels can increase saturated water content, aggregation, porosity, available water content, cation exchange capacity, and electrical conductivity.^10,13,6,16–21^

The dynamics of water within soil hydrogel matrices are not yet fully understood, as they depend on a complex interplay of factors, including soil type, bulk density, hydrogel dosage, and, under real conditions of repeated application of the input, the residual effect of active hydrogels. Thus, the method and amount (dosage) of application must be determined through soil-specific trials.^22^

A practical recommendation for using hydrogels in agriculture requires attention to their potential residual effects. For instance, the acrylamide-*co*-potassium acrylate hydrogel applied to sandy loam soil from a Brazilian semiarid region has proven effective in increasing water holding capacity and reducing the permanent wilting point, regardless of temperature or exposure time.^14^ Notably, the same study^14^ observed a persistent water retention effect, remaining evident even after 130 days. These longer-term residual effects are highly dependent on the specific type of polymer, biodegradability, prevailing climatic conditions, and water availability.

For safe and effective use of hydrogels in agriculture, a thorough understanding of the dynamics of water retention and/or release within different soil hydrogel matrices is essential. However, the influence of soil bulk density on these dynamics across varying polymer doses in such matrices is rarely addressed in the existing literature. Moreover, a comprehensive literature review has shown a significant gap: no rapid analytical method has been established for estimating the dose equivalence of active hydrogels, aimed at practical applications by farmers. Such a method is critical for assessing the residual effects of partially degraded polymers from prior applications and for recalculating appropriate doses for subsequent applications, as well as for the design of safe protocols for recurrent use of the input in short-cycle crops.

This study has two main objectives: (1) to investigate the influence of polymer doses in sandy clay loam soil hydrogel matrices with varying bulk densities on water retention and/or release, in order to guide optimized recommendations of hydrogel doses and (2) to develop a novel, time-efficient, and easy-to-use analytical method for estimating the dose equivalence of active hydrogels, aimed at addressing the residual effects of nondegraded polymers in practical agricultural applications.

A longitudinal time series study on hydrogel’s influence on water retention and release supports more accurate dose recommendations. In addition, a practical machine learning-based method, relying on a short time series, reduces laboratory time, allows flexible drying, and minimizes dependence on bulk density and moisture conditions. For a short-duration method, locally weighted regression (LWR) stands out among machine learning algorithms for its ability to effectively handle time series exhibiting pronounced localized patterns (short time series) in regression tasks.^31^

## Materials and Methods

### Experiment Location and Soil Properties

All experiments were carried out at the Embrapa Instrumentation laboratory in São Carlos, SP, Brazil. The soil type, located at geographic coordinates 21° 57′ 13.9″ S and 47° 51′ 10.9” W, is classified as dystrophic Red Yellow Latosol,^23^ corresponding to an Oxisol in U.S. Soil Taxonomy.^24^ Soil samples were collected from the 0 to 0.2 m layer, which is composed of 31.5% of clay, 5.7% of silt, and 62.8% of sand, classifying it as sandy clay loam texture.^25^

### Sample Preparation

The soil samples were sieved through a 2 mm mesh sieve and oven-dried at 100 °C for 24 h to obtain fine, dried soil. Cylindrical rings of poly­(vinyl chloride) (PVC) with a diameter of 75 mm and a height of 60 mm (volume of 244 cm^3^) were filled with the prepared soil. At the bottom face of each ring, filter paper (grammage of 80 g m^2^) and a nonwoven fabric filter were affixed, while a transparent plastic wrap was placed at the top. All components were secured with rubber elastics to prevent soil losses.

Hydrogel doses of 1, 3, and 5 g L^–1^ soil, referred to as H1, H2, and H3, respectively, were evenly mixed with the amount of sieved soil according to the volume of the PVC rings and the three soil density conditions (1.1, 1.2, and 1.3 kg dm^–3^ designated as D1, D2, and D3, respectively). Each PVC ring was carefully filled to accommodate the specific mass of the soil hydrogel matrix. The target bulk densities (D1, D2, and D3) were obtained by gently tapping the PVC cylinders with a metal rod until the desired apparent density was reached, as confirmed by the premarked height on the cylinder. Samples without hydrogel were also prepared (dose 0 g L^–1^ referred to as H0) for the three soil bulk densities (D1, D2, and D3).

### Hydrogel Synthesis and Its Characteristics

A nanocomposite calcium montmorillonite clay (MMt) hydrogel with a particle size less than 0.420 mm was used. It was synthesized using PAAm (polyacrylamide) and the biodegradable polysaccharide CMC (carboxymethyl cellulose), both obtained from Sigma-Aldrich. Components were formulated through the chemical polymerization of acrylamide monomers (AAm). The final composition of hydrogel was ([AAm + CMC]):m­(MMt) ratio (i.e., hydrogel mass per unit mass of clay) of 10:1. Hence, this hydrogel (10:1) contains about 10% of MMt.^26^

### Experimental Design and Analysis of Variance

A repeated-measure longitudinal design was employed with four treatments (H0–H3) applied across three bulk density levels (D1–D3). Soil water content (SWC) was recorded 12 times over 15 days to capture the longitudinal response of each density–treatment combination. Each condition was replicated four times, yielding 48 soil hydrogel samples. For analysis, the mean SWC of the replicates was used as the experimental unit. The mean of SWC measurements from replicates was taken as an experimental unit for each density-treatment pair.

Analysis of variance (ANOVA) was carried out using a linear mixed model,^27^ with fixed effects for the treatments and random effects for densities (groups). Intercept parameters from the linear regression models were used to test the statistical significance of the random effects.

The level of significance for all hypothesis tests was set at 5% (α = 0.05). The linear mixed model, ANOVA, post hoc analyses, and assumption tests were performed using the free statistical software Jamovi.^28^ The post hoc analysis for pairwise comparisons between treatments was a *t*-test with Bonferroni’s correction.^29^ Holm’s correction^30^ (also available in the Jamovi) was included just to check for agreement in decision-making between the two pairwise tests.

Two tests for the normality of residuals were applied: Kolmogorov–Smirnov and the Shapiro–Wilk tests to corroborate the assumption and check for consistency between both results. The intraclass correlation coefficient (ICC) was assessed using Jamovi’s implemented method for random effects based on intercepts. A high ICC value (ranging from 0 to 1) indicates that a substantial part of the variance can be attributed to soil density effects, meaning that random effects play an important role in explaining the observed differences in the outcome variable, which also supports the choice of a mixed linear model.

The method for estimating the model’s parameters was the restricted maximum likelihood (REML), which addresses the bias issue associated with maximum likelihood for variance components. The bound optimization by the quadratic approximation (BOBYQA) algorithm was selected for parameter optimization. Using Jamovi’s notation, the linear mixed model for optimization was given in the following equation:

$${\hat{Y}}_{\log(\mathrm{SWC})}∼1+T+H_{\mathrm{Dose}}+T:H_{\mathrm{Dose}}+(1+H_{\mathrm{Dose}}|D_{\mathrm{Soil}})$$ 1

where (the fixed effects): *Ŷ*_log(SWC)_ is the dependent variable, represented by the natural logarithm of SWC; 1 represents the intercept, which is the average value of Ŷ_log(SWC)_ when all other factors are zero; *T* is the effect of the factor time on Ŷ_log(SWC)_; the coefficient of this term will indicate how the natural log of water measurement changes on each passing days; *H*_Dose_ represents the effect of the applied hydrogel dose on Ŷ_log(SWC)_; the coefficient of this term indicates how the log of water measurements changes on average with each unit increase in hydrogel dose; *T*:*H*_Dose_ is the interaction effect between *T* and *H*_Dose_, indicating whether the effect of time on Ŷ_log(SWC)_ changes depending on the applied hydrogel dose; it shows if the change in water measurements over time varies based on the amount of hydrogel used and where (the random effects):

(1 + *H*_Dose_|*D*_Soil_) represents a random intercept for each soil density level (this means the intercept, i.e., average Ŷ_log(SWC)_ at baseline, can vary across different soil densities; additionally, the effect of hydrogel dose (*H*_Dose_) might also differ depending on the soil density).

The time factor (*T*) for modeling was coded as numerical values under the assumption of a linear fit with the natural logarithm of SWC. Thus, the SWC variable was transformed to a logarithmic scale (log­(SWC)). To corroborate the hypothesis of a linear trend, the Jamovi software provides the statistical significance (*p*-value < α) for the linear regression fitting.

### Soil Water Content in Mass Percentage

The total mass of each soil sample holder (PVC cylindrical ring, filter paper, TNT fabric filter, and transparent plastic film) was measured before the cylindrical rings were filled with soil samples. Hydrogel doses (H1, H2, and H3) were added to the treatment samples, all of which were conditioned to the three defined density levels (D1, D2, and D3), as detailed in the Sample Preparation section. Subsequently, all cylindrical rings were placed in a tray of water to allow the soil to be watered by capillarity. After 24 h in contact with water, the rings were removed, and the wet mass of each was measured using a balance (Bel Engineering/KL 20001). Soil water content was then assessed as

$$\mathrm{SWC}=\frac{\left(\mathrm{WM}-\mathrm{DM}\right)}{\left(\mathrm{DM}-\mathrm{RM}\right)}\times 100$$ 2

where SWC is the water content (%) in the soil hydrogel matrix; WM is the wet soil hydrogel mass (g); DM is the dry soil mass (g); and RM is the mass (g) of the cylindrical ring and other parts.

The increase in soil hydrogel matrix volume (swelling) was assessed by measuring the heights that exceeded the upper edge of each cylindrical ring at eight fixed points, equally spaced at 45° angles. The average of the heights at these angularly separated points was used to estimate the soil hydrogel volume expansion, as shown in the following:

$$\mathrm{MVE}=\frac{\sum_{i=1}^{8}h_{i}}{8\cdot hc}\times 100$$ 3

where MVE is the mean volume expansion (%); *h*_*i*_ is the height of the soil above the upper edge of the cylindrical ring (cm); and *hc* is the height of the cylindrical ring (cm). Note that the circular area of the PVC ring was excluded from eq 3, as it represents a constant in the experiment.

An ANOVA for significant MVEs was performed based on a randomized block design. The blocking was based on soil densities and therefore comprised three distinct blocks: D1, D2 and D3. The MVE ANOVA was performed only in the sample preparation stage (day 0).

The ambient temperature in the laboratory was acquired by using two 5TE sensors (Meter, USA) positioned less than 80 cm from the experimental table (one at each end of the table). The mean temperature recorded by these sensors was used as a representative measure of the air temperature (°C). A data logger (EM50, Meter, USA) was used to record air temperature on an hourly basis.

### Practical Method for Predicting Polymer Doses in Soil Hydrogel Matrices

The proposed analytical method for estimating active hydrogel doses in already conditioned soil hydrogel matrices prioritizes the minimization of procedural and time-consuming laboratory operations while ensuring significant results. The method consists of two phases, which are described in the following subsections.

### Predictive Modeling and Feature Extractions

LWR is a regression technique, also known as Loess, which fits a general function to data using a multivariate smoothing procedure. It locally fits functions of the independent variables by considering data similarity, resembling the concept of moving averages in time series analysis.^31^ This local fitting approach provides more representative estimates compared to traditional parametric functions like polynomials.^31^ Some LWR implementations use the entire data set with instances weighted by a weighting kernel (weighting function), while others use only a combination of a fixed set with *k* weighted instances, and (ii) it fits a regression model using the weighted instances to ensure local representativeness.

The advantage of LWR is that, with a well-defined similarity metric and weighting function, the model can more accurately reflect spatiotemporal conditions. Additionally, if the local fitting model allows some kind of extrapolation, LWR can extend estimates beyond the dependent variable (target variable) range of the training data.

In this study, multiple linear regression (MLR) was used for local fittings to simplify the analysis and provide some extrapolation capacity. The similarity metric (distance) was the Euclidean distance in a rescaled variable space, where each independent variable was normalized to a range of 0 to 1 (with 0 representing the minimum and 1 the maximum value). The LWR hyperparameters were optimized using a wrapper approach with cross-validation.^32^ The hyperparameters included the number of nearest neighbors (*k*, integer value) and the type of weighting kernel. The possible weighting kernels were linear, (1 – distance); Epanechnikov, 3/4­(1 – distance^2^); tricube, (1 – distance^3^)^3^; inverse, (1 + distance)^−1^; and Gaussian, (*e* ^distance^2^^).

The fundamental data set used for training and validation of the proposed method was extracted from all experimental data described in the Experimental Design and Analysis of Variance section. However, the original 15 day time series was reformatted into shorter series to align with the proposed simpler method that requires only 3 days of SWC measurements. This segmentation into a 3 day subseries was designed to emulate different consecutive SWC conditions and provide varied inputs for the model learning process, which underlies the flexibility of our proposal. Thus, the new data set with reformatted time series was used to train and validate the predictive model (LWR).

From the new 3 day series data set, a feature extraction process was performed to obtain four independent variables (features) for predictive modeling. This technique was chosen to capture temporal characteristics and enhance the predictive performance of the LWR model, following an approach analogous to that of Deng et al.,^33^ which aimed to improve classification accuracy in time series data. The extracted features included the initial water content (√SWC_*t*=24_) retained in the matrix and SWC losses over the three consecutive days, derived from the parameters of simple linear regressions (β_1_ and β*_1_). Additionally, the intercept parameter (β*_0_) was used as a reference for the predicted value at time *t* = 0. In summary, the regressors (extracted features) used to train the LWR model were

√SWC_*t*=24_: first measurement of SWC (day 1, *t* = 24 h)*,*

SWC ∼ *f*(*t*) = β_0_ + β_1_·*t*: 3 SWC measurements, for *t* = 24, 48, and 72 h*,*

√SWC ∼ f*­(*t*) = β_0_* + β_1_*·*t*: 3 SWC measurements, for *t* = 24, 48, and 72 h,

where *f*(*t*) and *f* *(*t*) are simple linear regressions fitted to 3 day series of SWC and √SWC, respectively, with *t* as independent variable (for *t* = 24, 48, and 72h).

The Weka data mining tool, version 3.8,^34^ was used to fit the LWR model and evaluate its performance. To ensure a more reliable and general assessment of the model’s performance, a 10-fold cross-validation method (10-fold CV) was implemented. This validation approach allows all data instances to be utilized as test sets across 10 LWR models trained using randomly selected instances in a mutually exclusive split (disjoint training and test sets), thereby providing a comprehensive evaluation of the overall performance of the method. The performance metrics were the root-mean-square error (RMSE) and the Pearson correlation coefficient (*r*) for all validation predictions (all test sets from a 10-fold CV).

Hyperparameter tuning was conducted using the wrapper implementation available in the Weka tool, specifically through the CVParameterSelection meta-classifier. The parameter range for the *k*-nearest neighbor algorithm was set to search for optimal values from 0 (where all training instances are weighted) to 100, in combination with the five aforementioned weighting kernels. To minimize the impact of random seed bias, the internal number of folds for CVParameterSelection was set to 100. Additionally, graphical analyses comparing actual hydrogel doses to predicted ones were employed to visualize the overall dispersion of all predictions.

Finally, two scenarios were evaluated: (1) the bulk density information was included as a regressor (independent variable) with its fixed values of 1.1, 1.2, and 1.3 kg dm^–3^; (2) the bulk density variable was excluded from the set of regressors to achieve a simpler and more flexible model, as proposed, which can operate effectively within a range of 1.1 to 1.3 kg dm^–3^ without requiring soil density information.

### Simplified Sample Preparation for Future Practical Applications

The proposed method was designed to operate with simpler and faster requirements for sample preparation. Thus, the laboratory procedure is a simplified version of that described in the Sample Preparation section. In total, it takes about 72 h from sample preparation to all gravimetric measurements and SWC calculations. As the proposal targeted a wide range of soil moisture, drying the sample for 24 h in an oven (100 °C) is not a mandatory procedure if the soil hydrogel matrix meets certain minimum conditions for sieving (2 mm mesh). The procedure to achieve the desired soil compaction does not require the establishment of fixed values; only bulk densities within the working range (1.1 to 1.3 kg dm^–3^) are necessary. As the proposal targeted a wide range of moisture levels in the sieved soil hydrogel matrix, it is sufficient to ensure that the initial SWC falls within a working range of 7.5 to 45.5%. While a more precise sample preparation method from the Sample Preparation section can be used for potentially better results, this simpler approach offers valuable information for practical farm management. In summary, the method only requires ensuring the following working ranges: ambient temperature between 22° and 28 °C; soil bulk density between 1.1 and 1.3 kg dm^–3^; and matrix water content (initial SWC) between 7.5 and 45.5% for initial SWC_*t*=24_. Thus, the proposed method offers significant simplification and flexibility in terms of operational requirements demanded by laboratory analysis, which enhances its potential for adoption in routine soil analyses.

Figure 1 shows a summary diagram that illustrates all processes involved in the proposed method, from simplified sample preparation to the machine learning model for predicting equivalent doses of the active hydrogel.

**Figure 1:**
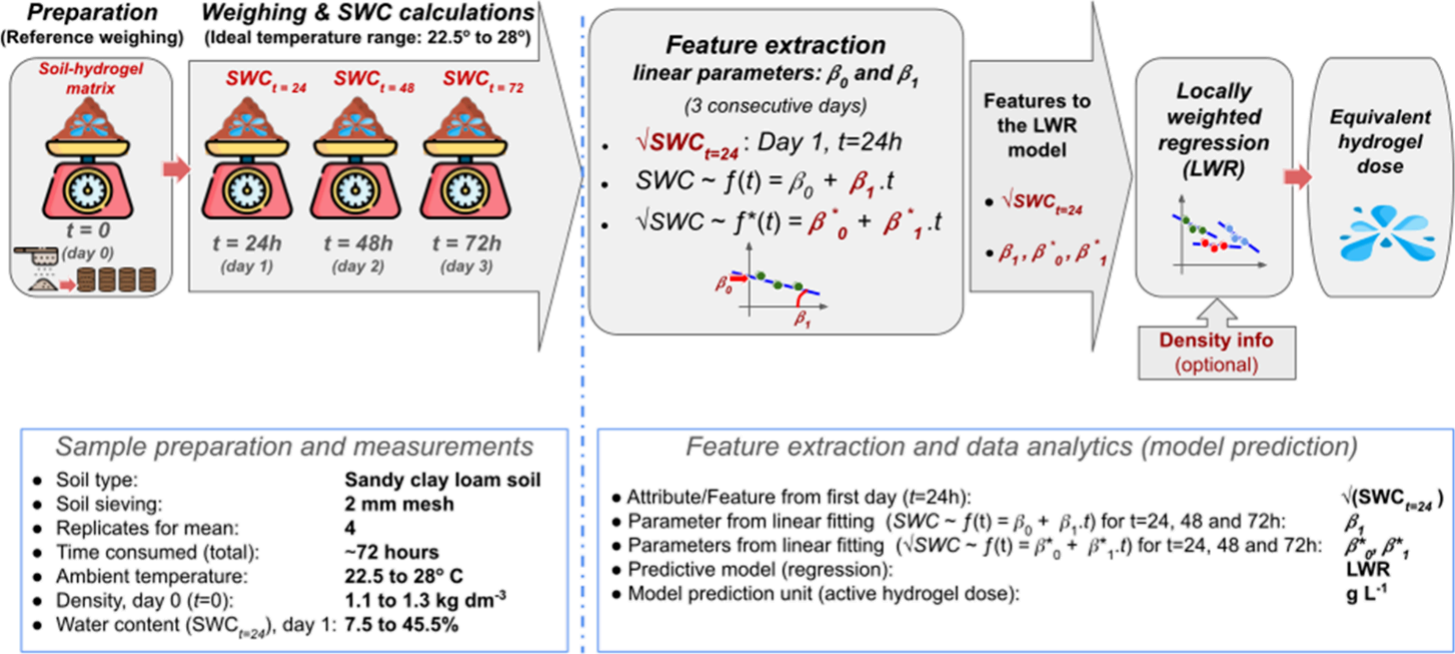
Summary diagram of the proposed method.

## Results and Discussion

The hydrogel dose of 5 g L^–1^ (H3) caused a significant MVE after water was added. Figure 2 illustrates four samples, with two of them (background) showing expansion caused by the H3 treatment.

**Figure 2:**
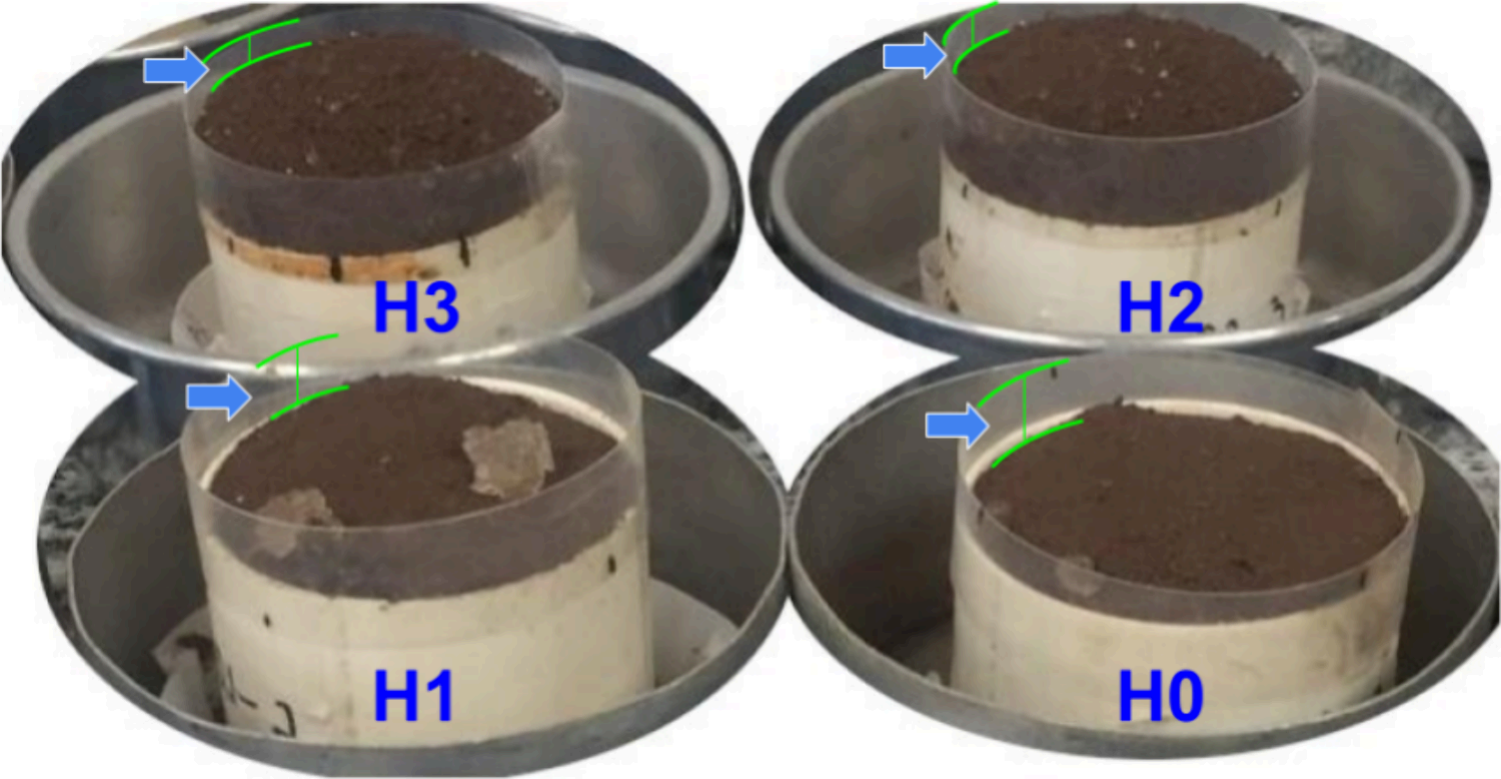
Expansion of the soil hydrogel matrices in the PVC cylindrical rings.

The ANOVA results for the MVE are presented in Table 1. The block effect (soil densities) was not statistically significant, likely due to the high variability among replicates and inherent MVE measurement imprecision (eq 3), which may have limited detection of density effects. In contrast, hydrogel dose had a significant effect. Post hoc comparisons using Tukey’s test (*p* < 0.05) showed statistically significant differences among all hydrogel doses. The mean MVE values increased progressively with hydrogel dose, reaching 13.91, 26.42, and 40.81% for H1, H2, and H3, respectively, confirming a dose-dependent response. Figure 3 provides a graphical summary of these findings.

**Table 1:** Analysis of Variance of the MVE (%)^a^

**causes of variation**	**degree of freedom (df)**	**mean square**	***F* test**	*p* **-value**
hydrogel dose (*H*)	2	2177.13	35.90*	0.0001
soil density (*D*)	2	58.32	0.96^ns^	0.3925
residue	31	60.50		
total	35			

a *, statistically significant; ns, not significant.

**Figure 3:**
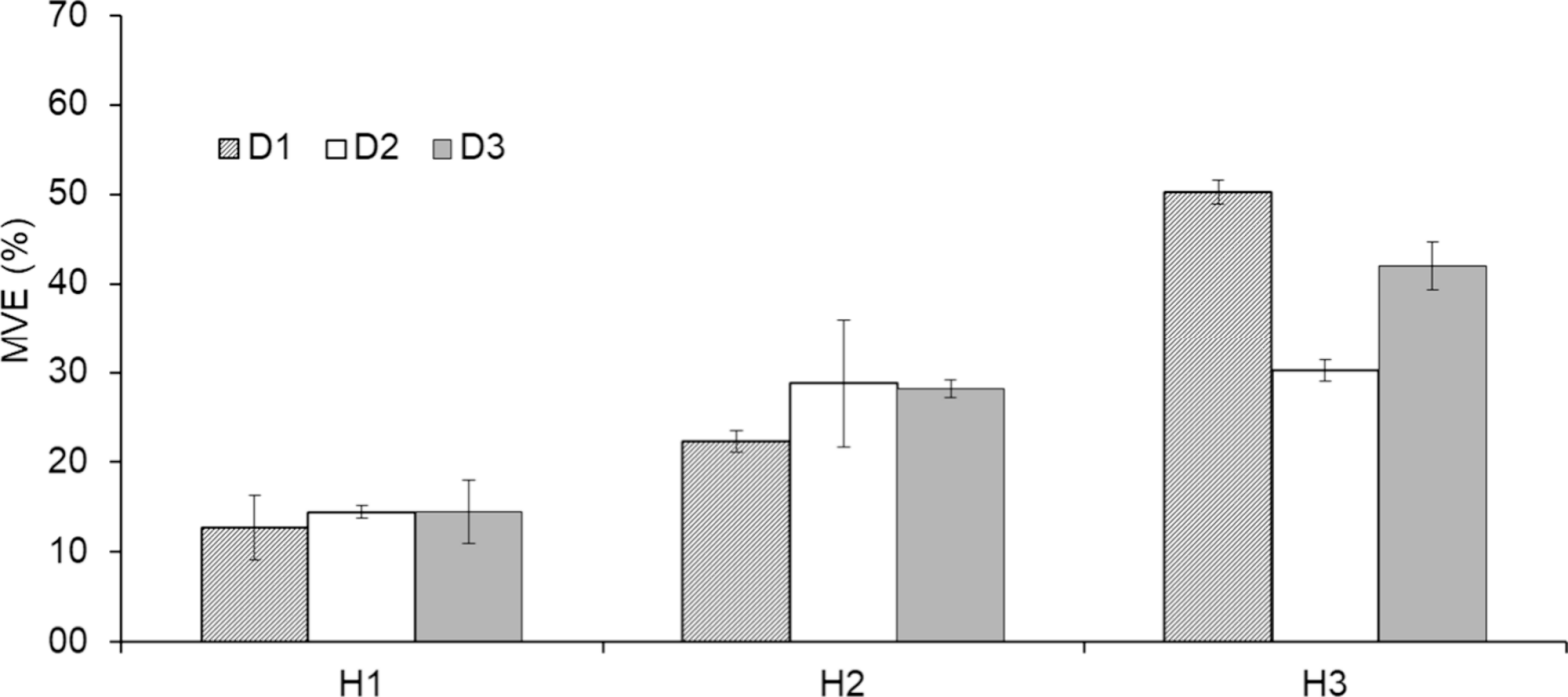
Mean volume expansion (MVE) of soil-hydrogel matrices (H1: 1 g L^–1^; H2: 3 g L^–1^; H3: 5 g L^–1^) for each soil density (D1: 1.1 kg dm^–3^; D2: 1.2 kg dm^–3^; D3: 1.3 kg dm^–3^).

### Analysis of Variance for Dose Effectiveness

The dynamics of water retention and/or release followed an exponentially decreasing pattern, influenced by the characteristics of the soil hydrogel matrix (Figure 4). Soils with higher bulk density tended to exhibit a more linear water content decline over time. This behavior is likely associated with reduced pore space, which limits water redistribution within the matrix, consequently restricting water flux and altering the release dynamics from the hydrogel.

**Figure 4:**
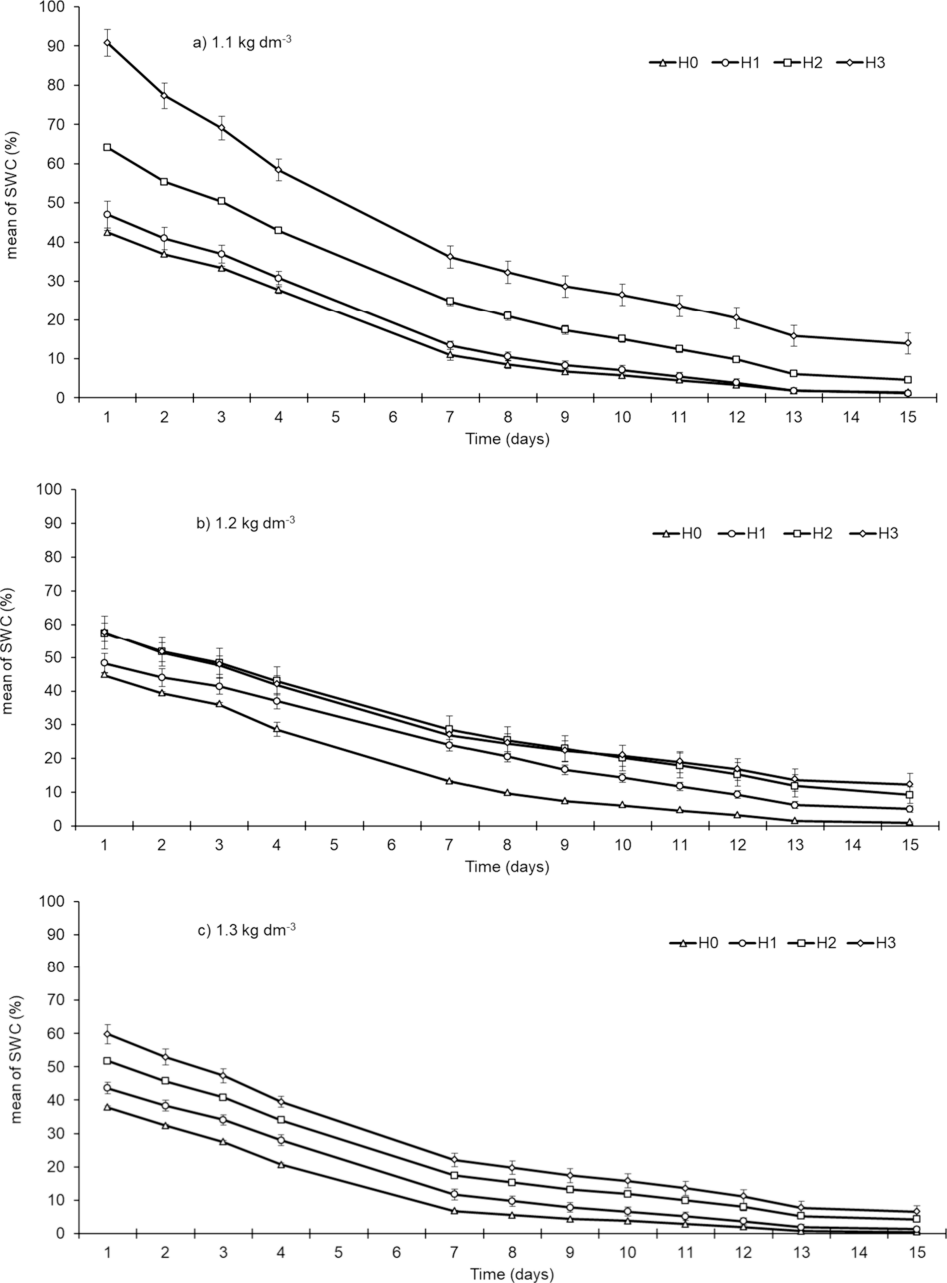
Dynamics of SWC as a function of time according to density and hydrogel doses (H0: 0 g L^–1^; H1: 1 g L^–1^; H2: 3 g L^–1^; H3: 5 g L^–1^).

The overall variance among hydrogel doses tended to increase as the soil density decreased. These results confirm the complexity of discriminating hydrogel dosages based on short-term SWC data, as density introduces distinct dynamics of water release over time. Even under fixed density conditions, this difficulty persists.

Fitting statistics of the linear mixed model using REML for all data are shown in Table 2. Statistically significant results were also observed for the linear trend (linear fit) of water release over time, as well as for the intercept, at the 5% significance level. The fixed model alone accounted for 91.7% of the variance, but adding the random effect raised *R* ^2^ to 98.2%, underscoring the value of a mixed-model ANOVA.

**Table 2:** Linear Mixed Model Fit by REML^a^

**fitting information**	**description**
model	*Ŷ*_log(SWC)_ ∼ 1 + *T* + *H*_Dose_ + *T*:*H*_dose_ + (1 + *H*_Dose_|*D*_Soil_ )
*R* ^2^ (marginal): fixed effect	0.917
*R* ^2^ (conditional): with random effects	0.982

a *R* ^2^ is the determination coefficient, which represents the variance explained by the model.

The global ANOVA for fixed effect (omnibus test) showed significant results for hydrogel doses (treatments) and the longitudinal factor (time), as well as the interaction between both (joint effect), at the 5% level (Table 3).

**Table 3:** Omnibus Tests for the Fixed Effect

**factors**	***F*-statistic**	**degree of freedom (df)**	***p-value***
time (longitudinal effect)	498.32^a^	11	<0.001
hydrogel doses (treatments)	401.33^a^	3	0.002
time x hydrogel doses (joint effect)	8.40^a^	33	<0.001

a Statistically significant.

Statistically significant results were also observed for the general linear trend (linear fit) of the log­(SWC) over time as well as for the intercept parameter at the 5% significance level. Consequently, water retention within the matrix followed an exponential decay, while water release over time, inferred from these dynamics, exhibited an exponential growth constrained by the initial water content. Changes that shape and particularize these general functions and the dynamics of water retention and/or release are highly dependent on the matrix density and dosage level of the hydrogel.

Figure 5a shows the general linear trend of the log­(SWC) over time, with the confidence interval (95%) of the mean as daily interval bars. Figure 5b shows the interaction between time and treatments with daily error bars representing the standard deviation. Differences between hydrogel treatments (H1, H2, and H3) and the control (H0) became increasingly pronounced over time. Repeated measures demonstrate both prominence and statistical significance across the 15 day period. However, trends in Figure 5b also highlight the challenge of dosimetric predictive modeling for active hydrogels over shorter time series, particularly at higher SWCs, where water saturation introduces significant confounding factors. Furthermore, the fixed-effects model alone did not reveal significant differences for all pairwise comparisons, emphasizing the need for a combined analysis that includes random effects. The parameter estimates and tests (*t-*tests) that reveal the significant impacts of the factors for the fixed-effects model are presented in Table 4.

**Figure 5:**
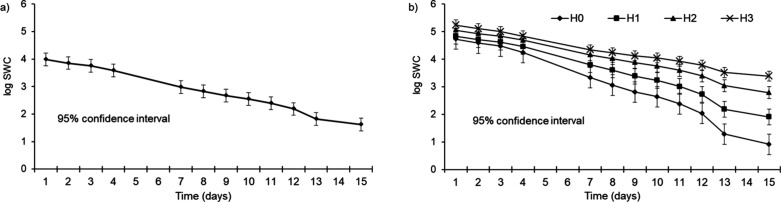
General dynamics of water content (SWC) within the soil hydrogel matrices: (a) an overall linear decay and (b) dose-dependent effects of hydrogel (H0: 0 g L^–1^; H1: 1 g L^–1^; H2: 3 g L^–1^; H3: 5 g L^–1^).

**Table 4:** Fixed-Effects Parameter Estimates

**factors**	**effect**	**estimate**	**SE**	**lower**	**upper**	**df**	*t* **-value**	*p-* **value**
intercept	(intercept)	3.98	0.12	3.73	4.21	2.39	32.61^a^	3.1 × 10^–4^
time	linear fit	–0.13	0.053	–0.23	–0.023	92	–2.40^a^	0.018

a Statistically significant.

The overall water release rate estimated for the fixed-effects model was −0.13 (log scale). In general, the average of water release rate over time for H0 across all densities was −0.236, which was 17.2% higher than H1, 50.2% higher than H2, and 79.1% higher than H3. On a logarithmic scale, the average water retention rate in the soil hydrogel matrix is estimated to increase by 15.47% for each g L^–1^ of hydrogel.

By adding the random effect to the linear mixed model, we obtained the ICC for the intercept, which was 0.7065. The tests applied to check the assumption of residual normality for the adjusted mixed model showed mutually agreeable decision statistics, with *p*-values of 0.481 and 0.143 for Kolmogorov–Smirnov and Shapiro–Wilk, respectively. Thus, there was no evidence to reject the normality assumption of the residues.

Significant differences in SWC mean values were found only between H0 and H2, and between H0 and H3 (Table 5). Therefore, considering the management and economic aspects in practical agriculture, H2 (dose of 3 g L^–1^) emerged as an optimal and low-cost recommendation for retaining water in different densities of a sandy clay loam soil.

**Table 5:** Post Hoc Comparisons: Comparing Pairwise Treatments (Hydrogel Doses)

**pairwise comparisons (hydrogel doses)**	**difference**	**SE**	*t* **-value**	*p-* **value (Bonferroni)**	*p-* **value (Holm)**
H0 × H1	–0.37	0.14	–2.59	0.73	0.26
**H0 × H2**	***–*0.82**	**0.063**	***–*12.91** ^a^	**0.035** ^a^	**0.035** ^a^
H0 × H3	*–1.08*	0.089	*–12.12* ^a^	0.04^a^	0.035^a^
H1 × H2	–0.44	0.090	–4.95	0.23	0.15
H1 × H3	–0.71	0.22	–3.14	0.53	0.26
H2 × H3	–0.26	0.14	–1.84	1.00	0.26

a Statistically significant.

### Predicting Hydrogel Active Doses

In the first scenario, where bulk density was included as an independent variable, the LWR model achieved a correlation coefficient (*r*) of 0.937 and an RMSE of 0.672 g L^–1^ based on a 10-fold cross-validation, supporting the model’s practical applicability. Figure 6 shows a scatter plot for visual comparisons of actual doses (*x-*axis) and those predicted by the LWR models. The method based on the LWR model exhibited homoscedastic errors in cross-predictions, although a slight loss of precision was observed at certain dose levels, likely due to experimental errors in sample preparation, ambient temperature fluctuations, and the high variance in SWC on the first day (*t* = 24 h). However, these factors do not statistically hinder the model’s ability to distinguish between significantly different doses, such as H0 × H2, H0 × H3, or even H0 and higher cumulative doses.

**Figure 6:**
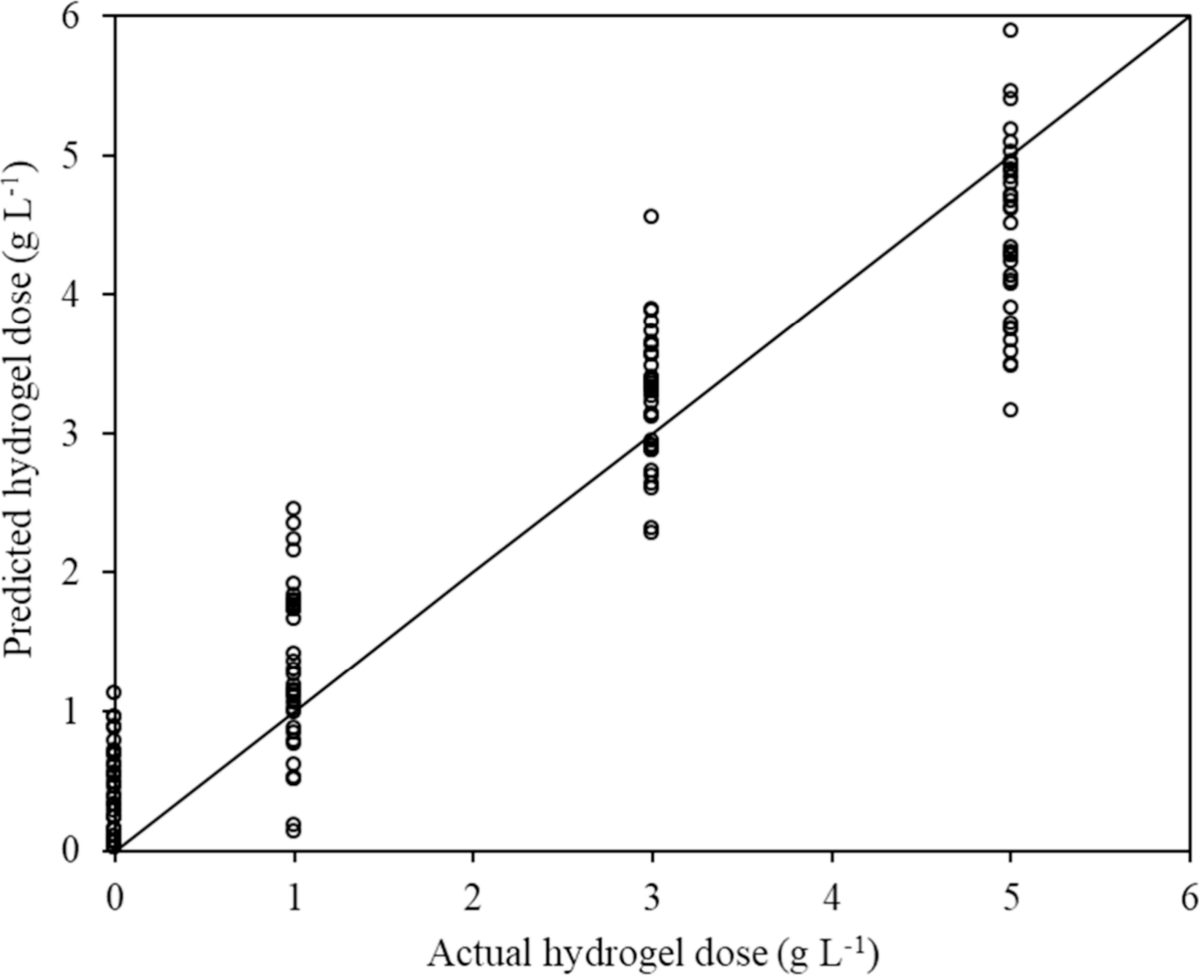
Scenario 1 (with bulk density): actual vs predicted hydrogel doses by the LWR model.

In the second analytical scenario, where the soil density variable was not included, some dispersion (imprecision) around the actual doses occurred in a 10-fold cross-validation (Figure 7). Despite a slight decrease in predictive accuracy (*r* = 0.875, and RMSE = 0.7486 g L^–1^), the LWR model still effectively distinguishes significant doses differences, such as between H0 and H2, H0 and H3 or even H0 and doses higher than 5 g L^–1^.

**Figure 7:**
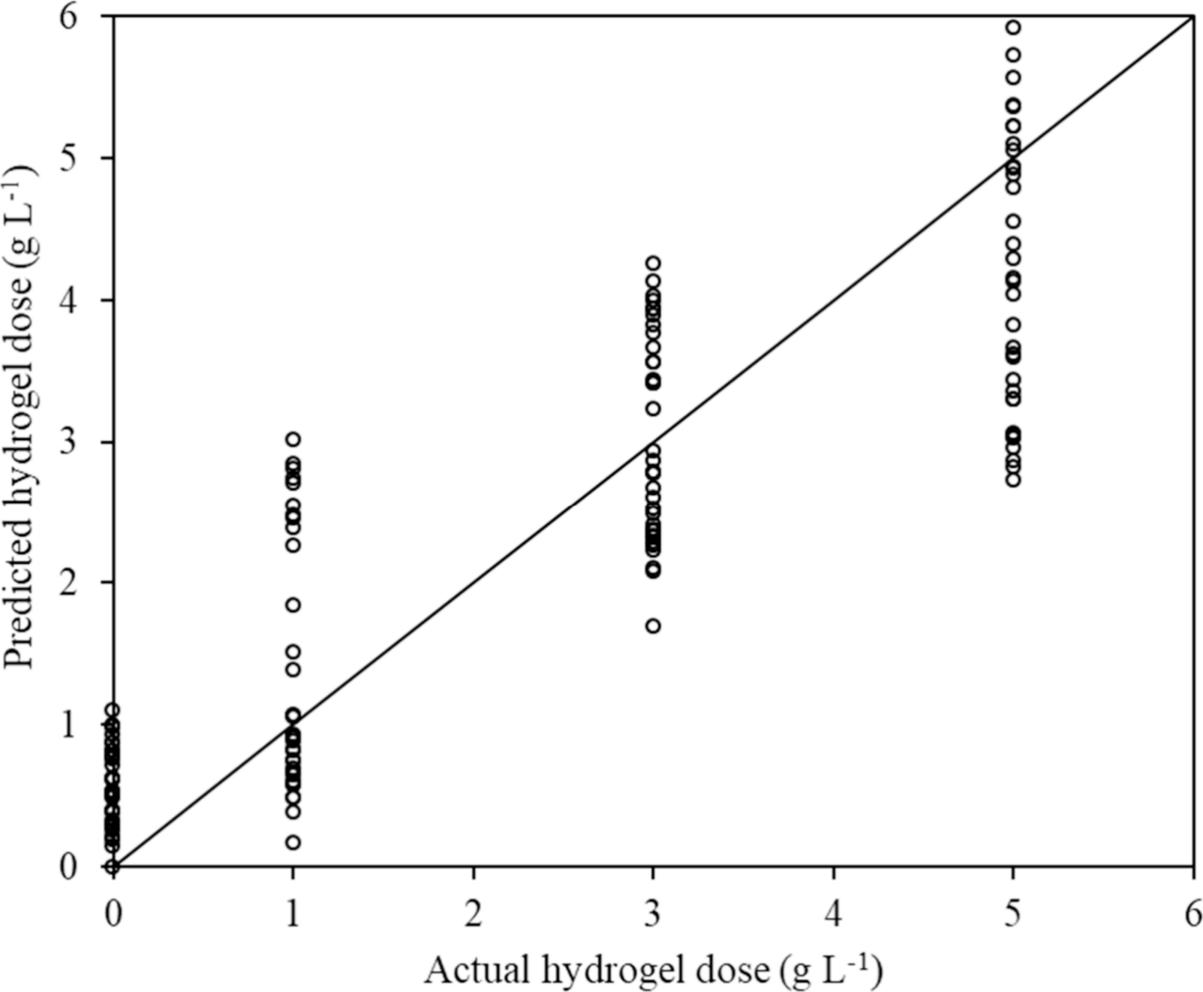
Scenario 2 (without bulk density): actual × predicted hydrogel doses by the LWR model.

The volumetric expansion was greater with the highest hydrogel dose (Figure 3). Nevertheless, some cracks in the soil hydrogel matrix structure were observed at this dose. Mendonça et al.^17^ noted that a 12 g dose of a hydrogel per 8 liter pot appeared disadvantageous due to soil cracking, as observed in this study with the 5 g L^–1^ dose (H3). Swelling of the soil pore matrix increases the space between pores due to the water-absorbing ability of hydrogels, while soil shrinkage changes the volume of soil matrix due to cracks. These movements of soil particles influence the distribution of pore sizes and affect the storage and movement of water, solutes, and air within the soil.^21^

The enhancement of soil water retention capacity through the addition of hydrogels has been reported in previous studies.^3,4^ While hydrogels are well-known to modify hydraulic properties, the specific dynamics of water within the soil hydrogel matrix are influenced by soil texture and, as demonstrated in this study, are also significantly affected by soil bulk density and hydrogel dosage.

SWC increased in clayey,^10^ sandy soils,^10,13,35^ sandy clay,^11,12^ and clayey soils^11^ when polymers were added. In clayey and sandy soils, water retention curves and SWC profiles after simulated evaporation deviated considerably from control values, with SWC rising as the dosage increased, especially in sandy soils. The average pore diameter expanded up to four times.^10^

The application of 4, 8, and 12 g of a potassium polyacrylate copolymer-based hydrogel per 8 liter pot filled with clayey soil increased water storage capacity by 12, 13, and 17%, respectively, which led to the recommendation of a 4 g dose.^17^ In contrast, our findings showed an optimal dose of 3 g L^–1^ for the nanocomposite calcium montmorillonite clay hydrogel. This demonstrates that dose recommendations depend on the type of soil (texture and bulk density) and the composition of the hydrogel. Regarding the latter, the increase in water holding capacity in different soil hydrogel mixtures depends on the water affinity and absorbency conferred by the hydrophilic functional groups present in these polymers, such as carboxyl, hydroxyl, and sulfonic groups.^8,36,4,37^

The introduction of a machine learning regression model for estimating active hydrogel doses in soil made the analytical procedure simpler, faster, and a flexible tool. To the best of our knowledge, there are no direct equivalents in the literature for comparisons. Cross-validation results revealed some prediction inaccuracies, notably when the bulk density variable (Scenario 2) was excluded from the LWR model training. The observed overlap in predictions for 3 and 5 g L^–1^ doses (Figure 6) suggests that the method may face challenges in differentiating between these two dosages. Conversely, both doses were clearly distinguishable from the control soil without hydrogel (H0), indicating that the model was able to detect cumulative effects of repeated polymer applications (residual effects) beyond 3 g L^–1^. Thus, the method enabled detection and qualitative classification according to dosage, suggesting its applicability as a screening tool for sandy clay loam soil hydrogel matrices. Furthermore, the LWR model can be trained with different soil types (varying textures), enhancing the method’s applicability to a broader range of conditions.

## Conclusions

The application of hydrogels substantially affects the soil water content (SWC) dynamics in sandy clay loam soil, which is modulated by the soil bulk density and doses. Among the treatments tested, the 3 g L^–1^ hydrogel dose demonstrated the best compromise between water retention efficiency and economic feasibility without compromising effectiveness across different soil densities. Statistical significance was established through the analysis of variance using linear models and post hoc tests, yielding a conditional *R* ^2^ of 98.2%, indicative of the model’s strong suitability when the fixed and random effects were considered. The recommended dose (3 g L^–1^) provides a practical starting point for improving water use efficiency in water-limited cropping systems.

The temporal pattern of SWC exhibited an exponential decay with significant interactions between hydrogel doses and time, underscoring the complexity of soil hydrogel interactions. An average increase of 15.47% in water retention rate (logarithmic scale) was observed per gram per liter of hydrogel applied under all treatment conditions. Although 5 g L^–1^ improved SWC, its effects were not statistically different from those detected with a 3 g L^–1^ dose. Furthermore, the dose of 5 g L^–1^ induced observable structural degradation in the soil hydrogel matrix, including visible cracking, which may impair soil integrity and plant establishment. Increasing hydrogel doses beyond 3 g L^–1^ not only escalates input costs but also may adversely affect the soil physical structure. These changes could have prolonged residual effects and impair subsequent crop cycles. Further studies are required to evaluate higher hydrogel doses under diverse field conditions and to assess long-term plant growth responses.

The LWR-based method, using a short-term SWC time series and incorporating soil bulk density, demonstrated high predictive accuracy (*r* = 0.937, RMSE = 0.672 g L^–1^), supporting its utility in field diagnostics. Even in the absence of bulk density data, the model maintained robust predictive accuracy (*r* = 0.875 and RMSE = 0.749 g L ^–1^), suggesting its potential for use in situations with limited soil data availability. However, model precision decreases under saturated soil conditions, where the feature space becomes indistinct (fog region), indicating that the approach is more suitable for unsaturated to moderately moist conditions.

These findings contribute valuable insights toward the sustainable optimization of hydrogel application in water-conserving agricultural practices. Future research should assess the generalizability of these results across different soil textures, climatic conditions, and extended cultivation periods to validate hydrogel efficacy and scalability in diverse agronomic contexts.
